# Promises and challenges of Blockchain in education

**DOI:** 10.1186/s40561-021-00179-2

**Published:** 2021-11-30

**Authors:** Jae Park

**Affiliations:** International Education (IE), The Education University of Hong Kong, Block D3, 1/F, Room 16, 10 Lo Ping Road, Taipo, New Territories Hong Kong

**Keywords:** Blockchain in education, Distributed ledgers, Learning, Rate of adoption, Sustainable development

## Abstract

Blockchain is arguably the next technology-mediated socioeconomic mega trend after the ongoing era of Net Neutrality and Big Data. This theoretical paper explores blockchain technology and its impacts on education. It is argued that we cannot take for granted that the network neutrality, popularized accessibility of the *Internet* and its influence on education will remain as we know it today. Blockchain promises, among others, a greater control over financing and investing in education, implementing instructional projects, a certification/accreditation system and learning. Education blockchain with its distributed ledgers would set novel standards of crypto-learning and crypto-administration that are acceptable across organizations and nations, enhancing thus the objectivity, validity and control of information without being compromised by socio-economic instabilities. The slow rate of adoption of blockchain technology in education reflects the rate in the fields of finance and management but, at the same time, it poses a few critical challenges such as lacking tangible incentives for technology maintenance or ‘blockchain mining’ (inward sustainability) coupled with a rather feeble orientation to collective development of education (outward sustainability).

## Introduction

Blockchain is one of many Distributed Ledger Technologies (DLTs) and it has been identified as a technology-mediated socioeconomic system that is taking over the era of Net Neutrality, Big Data, global market capitalization oligopoly and, broadly and aphoristically, ‘Google’s System of the World’ (Gilder, 2018). This paper discusses the actual and potential impacts of blockchain technology on education, that is, blockchain as a new paradigm for digital data management and learning.

As a time-honored social institution for knowledge transfer and a field of study, education has consistently been swift in adopting technologies from the industrial, financial and other sectors including the military. Education is undergoing a honeymoon period of Big Data, mass social media, STEM curriculum and data mining/analytics with subservient technologies such as Artificial Intelligence and Machine Learning. These are coupled, in turn, with refurnished teaching and assessment practices such as flipped classroom and technology-assisted evaluation. Some of these have been problematized, for example, Big Data for its critical issues of veracity (validity) and value (purposefulness) that are surreptitious as well as culturally invasive in the ways of obtaining and processing data such as mass surveillance, which pose tough ethical questions (Park, 2019).

Although the frontiers of veracity and value of Big Data are far from being clearly demarked, Big Data has been consistently relying on the principle of Net Neutrality. It might not take long, however, to see an end to it. In the book *Life after Google*, George Gilder (2018) argues that after the ongoing era of the open Internet, Big Data, and Google, blockchain is the next technology-mediated socioeconomic mega trend. Blockchain would be the new security structure of the Internet, which allows to keep for oneself and explicitly permitted others, a specific information with value.

This article explores blockchain technology and its impacts on education. It draws on the extant reports and research by those who wish to harness its potentials for education, from academics to international agencies in charge of monitoring education. The article starts with a general introduction to blockchain technology by arguing that its application in real life is recent, not yet widespread, and its evolution, unpredictable. It will be followed by a review of the extant literature on the actual use and potentials of blockchain technology in and beyond cryptocurrencies. The next section will be devoted to the potentials and actual applications of blockchain technology in and for education. The article ends with a twofold elaboration on why blockchain technology enjoys a slow rate of adoption by the education field.

## Backgrounds

### Evolution of Blockchain technology

Blockchain technology was originally intended for attesting the ‘who’, ‘what’ and ‘when’ of digital documents in 1991. Left unused for years, an incognito person with the pseudonym of Satoshi Nakamoto applied it into the first digital cryptocurrency in 2009—the *Bitcoin*. A decade into its application in real-life with cryptocurrencies, which are now counted by thousands, blockchain has already proven itself to be a technology that allows to secure digital data controlled only by its users. Even though its first broader application in cryptocurrency is considered as being technically secure, blockchain is subversive to the traditional financial system in the sense that it is not subject to third party scrutiny/attesting by financial institutions such as banks, credit card corporations, the Society for Worldwide Interbank Financial Telecommunication (SWIFT) system and state financial regulatory authorities.

Gilder briefly describes blockchain as:

A database, similar to a cadaster of real estate titles, extended to events, covenants, patents, licenses, or other permanent records. All are hashed together mathematically from the origin of the series, each record distributed and publicized on decentralized Internet nodes (2018, p. 241)

For the purpose of the present article, a discussion about the impacts of blockchain on education, a blockchain Block consists of: (1) The Data—its particulars and the Nodes taking part; (2) the Hash of the Block that is an identifier that gets changed with every change in the chain; and, (3) the Hash of the previous Block that constitutes a full logbook. An exception to the latter is the ‘Genesis Block’ since it is the first born.

Blockchain is antithetical to the Big Data due to its mode of creating, owning and sharing a dataset, for example, it is utterly against the Net Neutrality doctrine as it is enclosed and not free of cost at all. The core of blockchain technology is its heavily encrypted ‘Open Ledger’ or ‘Distributed Ledger’ (among a set or limited number of Nodes) through which, the veracity of a dataset is transparent to all Nodes within the same chain, and the latter is verified in a decentralized manner. For this reason, there must be a ‘proof-of-work’ or ‘blockchain mining’, which is the crucial task of verifying the validity of an incoming new Block by the ‘blockchain miners’. A ‘proof-of-work’, also known as ‘mining’, requires high speed and high energy-consuming processing and computing power, a service that has to be rewarded. A ‘proof-of-work’ task in the *Bitcoin* takes about 10 min and blockchain miners are rewarded with coins. As it will be detailed later, mining is one of the biggest technical challenges for blockchain technology in education.

Another interesting pre-determined arrangement over the process is ‘Smart Contract’ by which, under certain conditions stipulated digitally, specific procedures are executed by the power of peer-to-peer agreement. *Ethereum* is, for instance, a blockchain computer network platform that materializes smart contracts using a unique computer language (e.g., *Solidity*).

Although information such as smart contract and proof-of-work seem to be remote to the field of education of this article, these basic features are crucial for understanding and assessing the impacts of blockchain technology on education as will be argued later on.

### Blockchain in industries

The ultimate purpose of blockchain technology is avoiding uncertainties inherent to the authenticity of identities of people and the accuracy of shared information. This uncertainty avoidance is achieved by blockchain technology with shared information on virtual identity and transactions that are transparent to all parties involved yet, at the same time, meticulously veiled with powerful cryptography.

The number of potentially beneficial areas of social and economic life to which blockchain technology can be applied is daunting. Such potentials become materialized only when the users are able to diversify applications and move on from the current Blockchain technology 1.0 to the next level (Chen et al., 2018; Swan, 2015):

**Table:** 

Blockchain technology 1.0	Cryptocurrencies as a peer-to-peer cash payment system
Blockchain technology 2.0	Applications in stocks, bonds, loans, smart property, and smart contacts
Blockchain technology 3.0	Government, health, science, literacy, culture, and art

With the main focus on financial applications, Blockchain technologies 1.0 and 2.0 involve an explicit act of dodging regulatory interventions or refereeing by traditional third parties such as the state, banks and other international financial intermediary services. For example, in order to be legit, smart property’s transfer of a real estate ownership would always be publicly registered and subject to stamp duties in situ. In contrast, Blockchain technology 3.0 is envisioned as (1) applying the technology in areas beyond market capitals and finance, and, (2) ‘non-peer’ parties such as the state can partake in the creation of a distributed ledger as the genesis block or play a role as a Node, for example, in public service governance.

The potential areas for blockchain technology application are very broad, however, its actual use is still predominantly in finance (Gatteschi et al., 2020). Yet, even in finance, blockchain technology is migrating from 1.0 to 2.0, for example, when exchanging cryptocurrencies into and from ‘Fiat money’ (government-issued currency) or gold. As shown in the cases of *Gnosis®* and *Augur®,* when market predictions or oracles are accurately recorded in shared ledgers, they are rewarded monetarily. Other current applications of Blockchain technology 2.0 are in smart contract-based pension fund transfer services and money lending (Gatteschi et al., 2020, p. 98).

### Blockchain for public affairs

A non-financial use of blockchain technology is notary or comparable certification services that require arbitration in intellectual properties, copyrights and their licensing (Gatteschi et al., 2020). As alluded earlier in the history of blockchain technology, such certifications and time stamping were the original intended purpose of blockchain technology. Through smart contracts, it is possible to execute, for example, a last will with detailed conditions of inheritance transfer. Furthermore, public legal records such as marriage and divorce cases can be managed with blockchain. Smart contracts can also be used for real estate management, property transfer and pay-per-use as well as peer-to-peer insurance.

Blockchain technology is used not only for personal data management such as the authenticity of identities and personal information sharing but also by industrial sectors such as for construction logbooks, engineering works such as aviation and trades including, for example, tracking commodities such as diamonds, drugs and leisure event tickets (Gatteschi et al., 2020, p. 100). This has implications in education, for example, vocational and technical education, engineering education and training in trade and commerce. Used wisely and uprightly, blockchain technology can improve or resolve some dated side effects of human activities such as child labor and fair trade.

Blockchain technology could surely be applied for the benefit of the public and collective interests. Personal information management (e.g., immigration status), ownership (e.g., copyrights and housing) and payment records (e.g., tax and through smart contracts reducing the defaulting rate of bad loans). Blockchain technology applications that have already been tested in other fields can be used in the administration of the state and localities (Gloerich et al., 2020), which includes the education sector. Thus, an area of application still to be tested in real life is in the one-man-one-vote democratic electoral system with its ledger that is transparent to all political parties and constituencies. Similarly, blockchain technology can be used, tamper proof, in the administration of tax, mortgage, supply chain, delivery services, shipping, postal service and audits. It is not unthinkable, therefore, a few years from now, that undergraduate and postgraduate courses on blockchain could become a standard curriculum content for degrees in the areas of public administration and political science.

Another intensively reported area of blockchain technology application is in the healthcare system, both private and public. The healthcare system is one of the hardest fields where blockchain technology is being tested because it deals with problems that even money cannot solve—health threats and life quality. Traceability of health-related information is the main benefit of the blockchain technology in the field of public health. A meta-analysis of 197 studies identified five main application areas of blockchain technology in healthcare (Kassab et al., 2019): Managing and sharing healthcare records, medical supply chain, medical training and education, clinical research, and insurance claims.

Some other benefits of blockchain technology in healthcare are in the accessibility criteria of health records, transparency of health services, information security and better performance. There are also challenges of incorporating blockchain in healthcare, namely, (1) scalability and performance, (2) usability, (3) secure identification, and, (4) lack of incentives and willingness to adopt blockchain technology (Kassab et al., 2019, p. 11). Blockchain technology is being used “medicines, medical equipment, health supplies, besides reinforcing the value of health records and potentializing the ownership of the medical history to the patients through unified registers” (Kassab et al., 2019, pp. 12–13). At the individual level, sharing the medical record of a patient with her/his hash via smart contract provides clear-cut parameters for restrictions and access rights available to doctors and health professional across hospitals and even perhaps among nations in global health crisis such as the ongoing COVID-19 pandemic. Health wallet would be an added feature that can materialize hospital fees to the international purchase of vaccines. In drug administration, for example, pharmaceutical product distribution processes, from the producer to end users, can be better tracked by committing the parties involved to be responsible and accountable. In the future, blockchain could be a normal part of medical education, pharmacology students and nursing studies.

### Blockchain for grassroots initiatives

The most recent and people-oriented application of blockchain technology is for the socially responsible mode of production and crowd financing/accounting. It is distinctively a Blockchain technology 3.0, and it has been proving itself to be a driving force of future social organization. The crux of this blockchain technology is not the technology itself but its intended goals of social change and justice through peer-to-peer communication, autonomous organization and contribution to the globally distributed commons with shared innovative knowledge, resources and open cooperative economy called Commons-Based Peer Production (CBPP) (Bauwens et al., 2019). Blockchain technology is used to develop “ecosystems of collaborators based on distributed ledgers” (Bauwens & Pazaitis, 2020, p. 10), which, in turn, let a grassroots network of people to monitor and develop, for example, transnational territories and wildlife species such as the Amazonian rainforest and the African elephants. Among the extant projects, we have the *GainForest* for the Amazon rainforest and the *Great Elephant Census* for the African wildlife monitoring (Bauwens & Pazaitis, 2020). Training in blockchain could be a compulsory subject in the future for students in environmental studies and the lifelong learning of people who devote their lives for a sustainable world and nature conservation.

The foregoing discussion suggests that blockchain can serve as a tool to reify such post-capitalist socioeconomics despite the ongoing cryptocurrencies’ strong orientation towards a distributed capitalism. It is for this reason that proponents and practitioners of the CBPP advocates for a post-blockchain era (Pazaitis, 2020, p. 2):

[Distributed ledger technologies] have challenged the core assumptions of the financial and monetary system, opening up a discussion where these matters become relevant for an increasing fraction of society. Now, an ontological shift is necessary to break the chains of open innovation through CBPP. Post-blockchain encapsulates such a vision of a blockchain-informed transition that is not necessarily blockchain-driven.

Unlike blockchain-driven cryptocurrencies such as *Bitcoin*, a blockchain-informed CBPP empowers the grassroots, for example, digital wallets allowing them to do payments and receive money without a domicile or bank account. This process of empowering has a precedence in some developing countries where the state grants house and land ownership through housing/land reforms [e.g., Peruvian squatter dwellers receive an address which turn them to be economically active; see (Soto, 2002)]. The difference is that a blockchain-informed empowerment does not need a state intervention.

The discussion in this section demonstrates a tension in the use of blockchain technology. On the one hand, there is a hyper-capitalist use and usufruct of Blockchain technologies 1.0 and 2.0 whilst, on the other, a post-capitalist movement in blockchain that is based on peer-to-peer communication, resource commons and collaborative action. This somewhat paradoxical use of blockchain has important implications for education.

## Blockchain technology in and for education

Today, the mainstream perception of educational technology is usually through the prisms of Big Data, participatory social media, STEM education and data mining/analytics with subservient technologies such as Artificial Intelligence and Machine Learning. This context as well as pedagogic trend chiefly pertain to and depend on “Google’s System of the World,” which is, according to George Gilder, an awkwardly Marxist information structure that is being replaced by a new world order of distributed ledgers (2018). If the author of *Life after television* (Gilder, 1994) is correct, blockchain technology is a strong contender to be the most fundamental paradigm shift in digital communication and educational technology.

Much of the reported use of blockchain technology is, however, still at the level of Blockchain technologies 1.0 and 2.0. The *Project Connect* of the UNICEF is running, for example, a dozen of blockchain-mediated educational projects such as bringing open source technologies to developing communities, investment in educational projects, institutional funding, charity donations and crowdfunding (Cacioli, 2020). Across all of these applications, the main benefit is that records such as accounting, investments and donations to education cannot be altered or controlled by any single authority.

### Blockchain and educational administration

Blockchain technology can execute other tasks in education administration and management through its shared ledgers: School staff hiring via smart contracts, continuing professional development (Clark, 2016), performance and payments, from their first day of work to retirement pensions. Education institutions can apply blockchain technology to administer academic activities, for example, administering MOOCs all the way through actual degrees (Clark, 2016). A degree deserving course work could be executed:

smart contracts managed in blockchain systems could establish conditions under which a student would receive a certificate from a provider, and a series of those contracts could define a full degree program. As these students’ progress toward degree fulfillment, their blockchain records could be tracked automatically and shared in real time with potential employers. (McArthur, 2018, p. 3)

Sharples and Domingue report the University of Nicosia in Cyprus as the first university in the world to issue academic certificates with authenticity verification through the Bitcoin blockchain (2016). Authenticity verification could be within a single institution such as the reported case of Holburton School in San Francisco that uses an intra-institutional blockchain for authentication of certificates as well as among a consortium of education institutions (Clark, 2016). Furthermore, a national blockchain database of academic credentials could be created at different levels of education (ibid.).

Referring to a sizable international effort to lower barriers in favor of the global mobility of students and academics in the past two decades, Gatteschi and collaborators (2020) point out that blockchain technology could greatly contribute to a more effective and efficient recognition and management of certification and accreditation. Due to its immutability, transparency, and trustworthiness, blockchain technology can not only radically curtail degree frauds (Chen et al., 2018) but also monitor the mobility of students, educators and professionals. This can be done, say, through “some automatic solutions … which trigger a transaction as soon as a degree is earned, or an exam is passed” (Gatteschi et al., 2020, p. 106).

The scope of international administration of degrees and credit transfer would be way broader, more accurate and transparent than the extant regional mechanisms such as the Bologna Process, the Brisbane Communiqué and other few initiatives by the Association of Southeast Asian Nations (ASEAN) and ASEAN + 3 (China, Japan and South Korea) (Chao Jr, 2011; Dang, 2015). A crucial characteristic of blockchain technology in degree and accreditation management is its rescinding interventions and arbitration by third party organizations and government bureaus. A famous case was reported in an editorial column of a nursing education journal by Diane Skiba (2017): “MIT's Media Lab has been collaborating with the Learning Machine, which created a wallet app that allows users to have access to their blockchain-based credentials and add them to their digital resumes” (p. 220).

### Blockchain and learning sciences

As for learning and teaching proper, which is the main mission of education, the majority of recent literature continue to speculate on the potentials of blockchain technology rather than its actual application (Chen et al., 2018; Gatteschi et al., 2020; Sharples & Domingue, 2016; Zhong et al., 2018). Nevertheless, as early as February 2016, a Japanese technology company claimed to have successfully adapted blockchain technology to enable “open and secure sharing of academic proficiency and progress records” (Sony Global Education, 2016). Blockchain technology with its distributed ledgers of semi-public data could record learners’ acquired competencies, that is, an individual’s previous learning history stored on a publicly distributed ledger so that even the learner herself/himself is fully informed of her/his attained or lacking competencies (Gatteschi et al., 2020).

Chen et al. (2018) offer other potential future use of blockchain technology in actual teaching and learning. They suggest three areas of learning and instruction with greater potentials for gain: Formative assessment, learning activities design/implementation, and records of learning processes. A team of Hong Kong researchers (Zhong et al., 2018) proposed using the *Ethereum 1.0* blockchain platform for e-learning of vocabulary. However, their study is a conceptual modelling, hence, again, a potential application still to be tested. The study does not seem to be aware of the problem of proof-of-work of *Ethereum 1.0* whereas *Ethereum 2.0* that use a different technique called proof-of-stake is not mentioned.

An area of education that could potentially benefit the most from blockchain technology is learner evaluation and assessment (Clark, 2016). Here, students are the Nodes of a blockchain; their individual performance as well as peer evaluation of individual contribution to the group work in the context of collaborative learning, could be fairer, more efficient and safely stored (Chen et al., 2018).

Even at the level of modelling and conjecturing about education blockchain can sometimes pose challenges. The proposal by Chen and collaborators (2018) is that students’ learning can be attested in distributed ledgers, ‘crypto-learning’ so to speak, and disclosure of related information should be only among the partakers of the same blockchain and/or via smart contracts. Their suggestions are generally plausible yet with two glaring issues. First, their reduction of smart contracts into a contractarian tit-for-tat teacher-student relationship is rather questionable. If so, it could disrupt cultural sensitivities and local philosophies of education*.* Second, their suggestion of converting learning into money—digital coins or paper bank notes does not matter—and storing them in an ‘education wallet’ is even more problematic. The very act of monetizing learning and teaching not only would be an act of cultural invasion but also a clash against the almost universally accepted value of ‘learning to be’ (Delors & UNESCO, 1996).

### Blockchain and education as a (social) contract

Melanie Swan (2015) also describes human learning in contractarian terms: “Bitcoin MOOCs (massive open online courses) and smart literacy contracts encompass the idea of opening up emerging-market smart-contract learning to all individuals worldwide” (p. 61). Thus, almost every area of teaching and learning is a potential beneficiary of blockchain technology: “Overall, blockchain can be used to construct a balance to measure learning process and outcomes. It is a reliable and an equal proof of value for everyone” (Chen et al., 2018, p. 7).

A smart-contract learning linked to the utilitarian philosophy of ‘learn to be paid’ or ‘learn to earn’ has a number of technical and sociological complications. To start with, we need to keep in mind that the first and primus inter pares cryptocurrency *Bitcoin* is, in fact, not a coin or currency but, rather, a digital standard gold with a huge difference that its value can fluctuate far wider than gold. Thus, *Bitcoin* has been so far a hot arena for prey and predators of financial speculations. Thus, instead of naming blockchain-mediated education ‘Bitcoin learning’ amidst an early enthusiasm (Swan, 2015), it is probably more proper to use the term ‘smart contract learning’.

Based on the discussion in this section, signs are abound that blockchain technology has not been fully exploited in education. Most literature are about future possibilities of blockchain technology and claims of transferability of some specific aspects of it from various industries to education. Reported cases of actual application in education are significantly less and they are mostly pilot projects/experiments within one single or a few institutions. The main cause for this phenomenon is arguably neither a lack of need nor willingness of the education sector to use this emerging technology. The next section identifies a twofold cause of such a tepid technology adoption rate: (1) Technical constraints in implementation, and, (2) weak sustainable development goals.

## Challenges of Blockchaining education

### Technical constraints

There are important technical constraints for the adoption of blockchain technology in education. A first and perhaps the most challenging one is the proof-of-work, that is, the consensus mechanism for the verification of new blocks (see the Introduction section). The greater is the size of a blockchain, the bigger and more costly its proof-of-work (mining) gets. The scale and intensity of mining is simply mindboggling, for instance, *Bitcoin* miners compute about 450 thousand trillion solutions per second at a total cost in energy, machinery and maintenance labor of about USD 600 million back in 2015 (Watters, 2016). Due to its hyper-consumption of electricity, blockchain mining poses challenges to the climate change and China has become the first state to ban in all together (Tett, 2021). Mining a global blockchain in education will face the challenge of higher carbon footprint and “the solving of complex mathematical problems as part of the validation process without which fraudulent blocks could be added to the chain. So the transposition of mining into the context of the Blockchain Learning will require special consideration” (Devine, 2015, p. 4).

The problem does not end with the rewarding of the proof-of-work of miners. An education blockchain with students as Nodes, the rightful owners of their respective blocks, has additional challenges of *qualia*, that is, those aspects of learning that resist quantification:

If a student were to provide one of these [mining] services in return for a fee – how would the Blockchain manage the process...the technological mechanics of inviting participation, distributing assessments, collecting results and allocating rewards. (Devine, 2015, pp. 6–7)

An intra-school blockchain created, used and mined within one single education institution or small consortium is likely to be sustainably operated. A larger education blockchain at the national or global level, however, might be a *pointless* project as it is very likely to fail to exchange information in a decentralized community without having to go through several third parties. For example, university degrees are almost always granted by the state or university, which are third parties in a blockchain with student-Nodes.

Furthermore, Watters pointed out a fundamental ambiguity of an education blockchain where institutions are Nodes—trust:

discussions about ‘trust’ and the blockchain in education often frame students (and/as potential employees) as being untrustworthy – as lying about their degrees or their skills… The blockchain would purportedly verify those credentials. But it’s worth asking too if institutions are trustworthy. Which students, which institutions are and are not trusted? Why? By whom? What is actually the source of ‘trust’ in our current credentialing system?... How would the trustworthiness of blockchained credential-issuing institutions be measured or verified? (Watters, 2016)

An education blockchain would create a permanent record where a specific information cannot be changed or removed, that is, it remains immutable and unalterable to all Nodes involved. It has been questioned, however, whether this is really desirable for students themselves (Chen et al., 2018), or, whether a permanent record goes against the basic principle of education for growth, transformation and if not even redeeming/reinventing oneself with a second or third chance (Watters, 2016).

### Fragile alignment with sustainability

A second major cause of scarce real application of blockchain in education is, in my view, its lack of alignment with an authentic philosophy of sustainable development of education. This is a complex and multifaceted issue.

Although Audrey Watters considers herself to be the ‘Cassandra of Ed-Tech’, who tells the truth but nobody believes in it, I fully agree with her that education blockchain is still more of a hype today than a real solution of the many real life problems education faces. Instead of conjectures on potentials alone, we educationists who are interested in harnessing the power of this new technology should first ask a few teleological questions, “What problems can blockchain solve in education? What problems—technologically, ideologically—might the blockchain’s adoption in education create?” (Watters, 2016).

For one thing, the global cryptocurrency experience tells us that a piece of information in a blockchain might be immutable but not its value. The value of the same information fluctuates at the rate of human need and greed. We have to keep in mind that, in education, the value and usage of new pedagogies and technologies have many times engendered unintended consequences. To illustrate, the direction of the conceptualization of giftedness and high ability was set a century ago by Binet’s intelligence measurement and William Stern’s quotient, yet it unintendedly brought about a new form of social stratification—fitting people into a normal curve, assigning scores to their knowledge, determining social circles and ability-based institutional streaming (Park, 2016). Education blockchain with permanent records of student abilities and attainments could also produce a new form of social stratification that may well be worse and more universal than the case of the intelligence quotient. Ultimately, the type and degree of arbitrariness of institutional trust and value might hinder global efforts to build a sustainable future.

Despite the great potentials and efficiency of blockchain, without a clear philosophy of what is the goal of education and where we want to be, the question of “What problems can blockchain solve in education?” would remain unanswerable. It is plausible that blockchain empowers learners by granting ownership and control over their credentials (Schaffhauser, 2016) but it can also derail the purpose of education by generating a novel form of social inequality and inequity, or worsening the dated ones. Blockchain technology might turn schools, universities and related social institutions into an arena of social control and perpetuation of power, for instance, a permanent record in a fashion similar to the Social Credit System and mass surveillance could lead to serious privacy problems and oppression (Kostka, 2019; Snowden, 2019; Song, 2019).

This paper submits that, instead of serving hyper-capitalism or authoritarian social control of education and development, blockchain should ideally serve an education that prioritizes peer-to-peer cooperation and sustainability. The bottom line of education blockchain proposed by this paper is not the technology for the sake of technological novelty per se but, rather, education’s chief goals of social justice and sustainable development. Through peer-to-peer communication, autonomous organization and contribution to the globally distributed ‘education commons’ with shared innovative knowledge, pedagogic resources and open cooperative education systems (see Bauwens et al., 2019). In other words, blockchain would have given a far better use with a clear philosophy of a sustainable and decentralized development of education where the main goal lies beyond the frontiers of bureaucratic efficacy, academic credentials and the ‘learning is earning’ sort of monetary enticements cum social control.

In what constitutes the most recent changes in the world of blockchain, Gillian Tett (2021) reports that the total value of the cryptocurrencies market of USD 1.5 trillion is now under scrutiny and retaliation as governments and banks try to counter cryptocurrencies’ influence. In June 2021, the Bank for International Settlements (BIS)—the mother and arbiter of all banks including central banks—stated: “Innovations such as cryptocurrencies, stablecoins and the walled garden ecosystems of big techs all tend to work against the public good element that underpins the payment system” (as cited in Tett, 2021) and BIS proposed ‘central bank digital currencies’ as a solution (Tett, 2021). In fact, the *stablecoins* themselves were launched by commercial institutions in their attempt to counter the instabilities generated by cryptocurrencies, and by pegging *stablecoins* to sovereign currencies and equivalent standards. *Ripple®* is, as an alternative, a far more practical and agile application of blockchain technology since it works with a limited number of international banks that form a consortium and, not without paradox, to make transactions without paying bank service charges. What is relevant for education is that education blockchain is not invulnerable or immune to regulations by external forces.

## Conclusion

Blockchain is considered as the next technology-mediated socioeconomic mega trend after the ongoing era of Net Neutrality and Big Data (Gilder, 2018). It promises to provide humankind with a Cryptocosm *cum* Life 3.0, hence, regarded as an all-encompassing and disruptive technology. This paper explored blockchain technology and its impacts on education.

The extant literature on harnessing its potentials for education speculate on the prodigious potentials of this technology. They conjecture on how industrial and financial blockchain can be adopted by education, and across almost all of its subdomains such as financial procurement, school funding, donations, school payroll, teacher professional training, human resource management, cross-border academic credit recognition, certification and degree transfers, monitoring students’ learning and attesting their actual capabilities.

Blockchain with its distributed ledgers certainly promises greater efficiency and control over educational administration and management. However, its real life application is almost exclusively reduced to identity authentication, degree certification attesting and a few cases of cryptocurrency-mediated monetary transactions in terms of school fees and donations. Two main causes of this lagging adoption rate of blockchain in education have been identified and discussed, namely, the technical and philosophical constraints.

Technically, blockchain is yet to be adopted by the mainstream industries due to its scalability issues and still limited technical familiarity and skills (Gatteschi et al., 2020). The phenomenon of an even smaller number of applications in education is, it was argued, due to the problem of proof-of-work, which is greater, the bigger is the number of blockchain Nodes. It is not possible to find any real life example of massive and global education blockchain at a comparable scale of cryptocurrencies (in millions). There is simply no clear and appealing incentive and motivation for performing its proof-of-work although this situation might change with the upcoming merging of *Ethereum* 1.0 and *Ethereum* 2.0 with a new technique of verification called ‘proof-of-stake’ expected to occur in 2022.

Philosophically, existing education blockchain usages tend to be excessively pragmatic in trying to give solutions to a limited range of administrative and managerial tasks, and within an institution or within small consortium of institutions at most. More glaringly, education blockchain lacks a robust philosophy of education aligned with sustainable development. It was suggested that perhaps a cooperative-common sort of education blockchain where resources are shared for collaborative and sustainable development might be more salutary for our common future, and it should be not only for the global metropoles but for all open societies.

To conclude, blockchain in and for education is a plausible forecast of an upcoming all–encompassing mega transformation yet, as a technology, it is subject to the principle of magnification and reduction, that is, “For every enhancement of some feature, perhaps never before seen, there is also a reduction of other features” (Ihde, 1993, p. 111).

## Data Availability

Not applicable for this review paper.
